# Development and Validation of a Passive Surveillance System for Early Detection of Pepino Mosaic Virus in Commercial Greenhouse Facilities

**DOI:** 10.3390/v18080866

**Published:** 2026-08-07

**Authors:** Hester Roberts, David W. Waite, Subuhi Khan, Stella Veerakone, Joe Tang, Jeremy R. Thompson

**Affiliations:** 1 Plant Health and Environment Laboratory, Ministry for Primary Industries, P.O. Box 2095, Auckland 1140, New Zealand; roberts.hester@gmail.com (H.R.); subuhi.khan@mpi.govt.nz (S.K.); stella.veerakone@mpi.govt.nz (S.V.); joe.tang@mpi.govt.nz (J.T.); 2 Kākano Biosciences, Half Moon Bay, Auckland 2012, New Zealand

**Keywords:** pepino mosaic virus, PepMV, environmental RNA, surveillance, detection, PCR, high-throughput sequencing, virology

## Abstract

The use of environmental nucleic acid (eNA), both DNA and RNA, as a means for surveillance has been a fixture in the scientific literature for many years. The application of environmental screening for genomic signatures of organisms of interest—particularly those of diagnostic concern—is a promising yet still under-utilised tool for sample screening. While the literature tends to focus on the use of high-throughput sequencing (HTS) to detect organisms of interest using metagenomic or metatranscriptomic sampling, this approach is not cost-competitive with more traditional targeted molecular test methods. Consequently, eNA sampling still has not gained significant traction in practical settings despite its popularity in ecological research. To address these issues in a biosecurity context, we report here the development of a testing protocol to monitor irrigation water for the presence of pepino mosaic virus (PepMV) that also includes an endogenous *Sphingomonas* control. We employed passive sampling through the immersion of filtering devices into the water system at three commercial growing operations at two time points to collect samples with minimal hands-on effort, while simultaneously developing and validating molecular methods for the recovery of RNA competent for both PCR and high-throughput sequencing. We demonstrate not only the ability to detect PepMV from water collections, but also that the method is robust to the accumulation of non-target material and does not lose signal if viruses are only transiently present in the water system. Finally, we developed a capsid-integrity pre-treatment protocol for differentiating between intact and denatured (non-viable) virus particles during PCR testing. This work presents a low-cost and low-effort technique for proactive screening of commercial greenhouse facilities to facilitate early detection of harmful crop pests and pathogens.

## 1. Introduction

Environmental nucleic acid (eNA) testing has become an immensely powerful tool across biodiversity monitoring, invasive species detection, and conservation science [1,2,3]. It enables sensitive, efficient, and non-destructive detection of organisms from genetic material shed into the environment. Research shows that eNAs can outperform traditional survey methods such as visual searches, trapping, or netting, particularly when species are rare, cryptic, or present at low densities [2,3,4]. This makes eNAs invaluable for early detection and ecosystem management. Its utility spans aquatic, terrestrial, and even airborne environments, allowing researchers to non-invasively track any living species. Studies also highlight eNAs’ effectiveness in identifying endangered or invasive species and enhancing biodiversity assessments, with metabarcoding proving especially transformative for detecting rare taxa and characterising entire ecological communities [2,3,4,5,6,7].

Although less studied, the environmental surveillance of viruses reached prominence during the COVID pandemic [8,9,10] and became a key tool globally to track and manage the spread of the disease. Learnings from this approach are now being applied to other viruses like Mpox [11], and Influenza [12,13]. In Aotearoa New Zealand (NZ) and globally, wastewater testing became a key tool for identifying COVID outbreaks, monitoring disease trends, and identifying variants, demonstrating how eNAs can inform timely public health responses and complement traditional clinical surveillance frameworks [14,15].

The application of eNA testing for plant viruses has also been demonstrated. Lopez-Roblero et al. applied an eNA sequencing approach to determine viral pathogen diversity in wastewater, freshwater, and sludge from Mexico, detecting fifteen different phytopathogenic species [16]. Similarly, metatranscriptomic sequencing of freshwater rivers in South Korea and farm irrigation systems in Slovenia identified both water bodies as reservoirs and potential transmission pathways for well-documented plant viruses [17,18]. The transmission risk in the case of Tomato brown rugose fruit virus (ToBRFV) was reported by Mehle et al., who showed that the release of virus particles from the roots of infected plants can act as a source of infectious ToBRFV particles in irrigation water [19].

In April 2021, pepino mosaic virus (PepMV; species *Potexvirus pepini*; family *Alphaflexiviridae*) was first detected in NZ in a South Auckland commercial greenhouse [20]. To improve greenhouse phytosanitation during ongoing management, we began testing unenriched irrigation water from affected and unaffected facilities using targeted reverse-transcription quantitative PCR (RT-qPCR) [20]. PepMV is a positive-sense single-stranded RNA virus that was first identified in 1974 in Peru infecting pepino plants (*Solanum muricatum*) [21]. It later became recognised as a pathogen of tomato (*S. lycopersicum*) in 1999 when it was identified in greenhouses in the Netherlands [22]. Since then, PepMV has emerged as an endemic pathogen significantly impacting tomato crops worldwide. Symptoms characteristically include chlorosis, fruit marbling, leaf blistering and necrosis [23]. Symptom expression is affected by various factors—environmental conditions, tomato cultivar and virus genotype—with the latter having the potential to switch from a mild to an aggressive phenotype due to a single-nucleotide substitution [24]. PepMV is highly infectious and is readily transmitted by mechanical means and spreads quickly via plant-to-plant contact. Water is also a source of PepMV infection [25].

The establishment of PepMV within New Zealand teaches a powerful lesson: that incursions of new plant viruses must be detected early if containment and eradication are to be viable options during disease response. With PepMV now firmly entrenched in the country, and the recent detection of ToBRFV in Australia [26] the need for proactive monitoring of growing facilities is greater than ever before. We identified eNA monitoring of greenhouse water systems as a potential cost- and labour-efficient option to achieve this goal. Irrigation water is often recycled within facilities and so has the potential to form a self-enriching reservoir of organisms within the facility.

As irrigation is performed over areas of plants, collecting water from the population avoids the need to sample each individual plant within a growing unit for comprehensive crop coverage. Irrigation or drainage water can be collected on site and then filtered, centrifuged, or otherwise concentrated for testing. This approach has been popular in recent years for monitoring the spread of SARS-CoV-2 in wastewater systems [27,28]. While this approach is effective, processing large volumes of water for laboratory testing does not scale well. This approach captures only a snapshot of the water column contents at the time of testing and, when monitoring for rare event detections, may not be robust if the microorganism load fluctuates with time. To overcome these limitations, a passive sampling approach [29,30] can instead be deployed for water monitoring. Under a passive sampling regime, a bio-adherent material can be immersed in the water system for a period during which it captures and accumulates biomass from the water column [31]. This material can then be taken to the testing laboratory and treated as a source of eNAs for undergoing nucleic acid extraction and standard molecular testing protocols.

In this study, we aimed to refine the existing crude PepMV testing of unenriched irrigation water taken from horticultural greenhouses by incorporating and optimising site sampling, sample enrichment via passive sampling, and detection methods. We applied metatranscriptomic analysis to greenhouse irrigation water microbiomes to identify suitable organisms for use as endogenous sample controls. To discriminate between active and inactive virus RNA, we applied a chemical modification using platinum chloride to prevent PCR amplification of RNA in compromised viral particles or RNA circulating freely in the water system. Our results provide a framework to operationalise agricultural water testing for the early detection and monitoring of endemic and invasive viruses of agricultural importance.

## 2. Materials and Methods

### 2.1. Plants and Virus Purification

Plants of *Nicotiana occidentalis* 37B were grown in soil or rockwool under greenhouse conditions of 18 ± 3 °C, 16:8 h light:dark, and 60% relative humidity. Crude sap from PepMV-infected plant material was inoculated onto *N. occidentalis* 37B indicator plants at a 4–6-leaf stage using a previously described method [32]. Two to three weeks later, symptomatic leaves were harvested and PepMV virions were isolated as previously described [33], and purity assessment was performed by polyacrylamide gel electrophoresis. Viral quantification was performed using reverse-transcription quantitative PCR (RT-qPCR) against a synthetic gBlock containing the target region under control of a T7 promoter sequence (Integrated DNA Technologies, Coralville, IA, USA). Target RNA was transcribed using the HiScribe T7 Quick High Yield RNA Synthesis Kit (New England Biolabs, Ipswich, MA, USA), then DNA was removed and RNA was purified with the Monarch RNA Cleanup Kit (New England Biolabs). The resulting RNA was quantified using the Qubit RNA High Sensitivity Kit (Invitrogen, Carlsbad, CA, USA) and used to construct standards for absolute quantification. RT-qPCR primers, probes, and reaction conditions are described below.

### 2.2. Quantitative PCR Detection of Target Organisms

RT-qPCR reactions were performed to detect circulating RNA of three molecular targets across the course of these experiments—PepMV, Kyuri green mottle mosaic virus (KGMMV), and luciferase. Reactions were performed in a 20 µL reaction mixture containing the qScript XLT 1-Step RT-qPCR ToughMix (Quantabio, Beverly, MA, USA) and 1% DMSO. PCR primers and probes were as previously described for PepMV and luciferase targets [34,35] with the following cycling conditions: reverse transcription for 15 min at 50 °C, then initial denaturation for 2 min at 94 °C, followed by 40 cycles of denaturation at 94 °C for 10 s and a combined annealing/extension at 60 °C for 30 s. Testing for KGMMV was performed using an in-house assay targeting a 104-nucleotide span at the 3′ end of the replicase gene. Cycling conditions were like those above, but with a 45 s annealing/extension step per cycle.

We also measured the accumulation of DNA from the bacterial genus *Sphingomonas* using a genus-specific qPCR assay [36]. Reactions were performed in a 20 µL reaction mixture containing the SsoFast EvaGreen Supermix (Bio-Rad Laboratories, Hercules, CA, USA) and 10 µM forward and reverse primers. Cycling conditions consisted of initial denaturation for 2 min at 95 °C, followed by 42 cycles of denaturation at 95 °C for 10 s and a combined annealing/extension at 62 °C for 10 s. After cycling, melt curve analysis was performed across a temperature range of 95–65 °C with 0.5 °C decrements in temperature, holding for 5 s at each step.

### 2.3. Comparative Assessment of Filter and Nucleic Acid Extraction Techniques

Filter membrane products were assessed for their ability to capture target viruses and their amenability to nucleic acid extraction (Table 1). Filters were spotted with 100 µL of water carrying three molecular targets at differing concentrations: 1 million PepMV virus particles, 50 pg luciferase RNA (Promega, Madison, WI, USA), and 50 pg of DNA from a gBlock (Integrated DNA Technologies, Coralville, IA, USA) construct encoding the conserved 3′ noncoding region of KGMMV [37]. Polyvinylidene fluoride (PVDF) filter membranes (Merck KGaA, Darmstadt, Germany) were activated by treating with a 15 s exposure to methanol prior to use for all tests below, as per manufacturer’s instructions.

Total nucleic acid extraction from each filter membrane was performed using three different methods. In each case, approximately 4.3 cm^2^ of membrane was excised and homogenised in 2 mL of lysis buffer. The homogenate was then centrifuged at 6000× *g* for 1 min, after which the supernatant was removed and purified using a nucleic acid purification kit. For the first method (RLT method), samples were homogenised using a Homex grinder (Bioreba, Reinach, Switzerland). Total nucleic acids were then extracted by the RNeasy Plant Mini Kit (Qiagen, Hilden, Germany), using the RLT lysis buffer without β-mercaptoethanol, and eluting a final volume of 50 µL. The second method (CTAB method) homogenised membranes in 2 mL cetyltrimethylammonium bromide (CTAB) buffer using a Homex grinder. The CTAB buffer consisted of 2.5% (*w*/*v*) CTAB, 100 mM Tris-HCl, 50 mM EDTA, 1.4 M NaCl, and 1% (*w*/*v*) PVP-40. The centrifuged supernatant was then purified using the InviMag Plant DNA Mini Kit (Invitek, Berlin, Germany), which has previously been reported as capable of purifying high-quality RNA from plant extracts [20,38,39], eluting a final volume of 100 µL. The final extraction method (GH+ method) applied bead beating to the membrane, in a lysis buffer of 6 M guanidine hydrochloride, 0.2 M sodium acetate, 2.5% (*w*/*v*) PVP-40, and 25 mM EDTA. Following lysis, 5 M DL-dithiothreitol was added to the homogenate to a final concentration of 0.15 M, and samples were incubated at 65 °C for 15 min. The centrifuged supernatant was then purified using the InviMag Plant DNA Mini Kit as above. Purified nucleic acids from each filter/extraction combination were assessed for their sensitivity and reliability by performing RT-qPCR for PepMV, KGMMV, and luciferase.

### 2.4. High-Throughput Sequencing and Bioinformatic Analysis

Irrigation water was collected from three commercial growing facilities in two sampling efforts and prepared for high-throughput sequencing (HTS). Biological material in the water column was concentrated using Nanotrap Microbiome A particles, in combination with the QIAamp Viral RNA Mini Kit (Ceres Nanosciences Inc, Manassas, VA, USA), according to manufacturer’s instructions with a modified final elution step. Purified RNA was eluted from the spin column using RNase-free water instead of Buffer AVE. A 50 µL volume of RNase-free water was added to the spin column and incubated for 1 min before centrifugation. This process was then repeated with a second volume of 50 µL RNase-free water, yielding a final volume of 100 µL.

Eluted nucleic acids were then assessed for quality via Nanodrop and quantified using the Qubit RNA HS Assay Kit (Thermo Fisher Scientific, Waltham, MA, USA). DNA was removed from the extract using the RapidOut DNA Removal Kit (Thermo Fisher Scientific), then RNASeq library preparation was performed using the Zymo-Seq RiboFree^®^ Total RNA Library Kit (Zymo Research, Irvine, CA, USA) as per manufacturer’s instructions. The resulting libraries were sequenced on the Illumina MiSeq system with a 2 × 250 bp Paired-end V2 MiSeq Reagent Kit (Illumina Inc, San Diego, CA, USA).

Raw sequence reads were trimmed for quality using fastp (v0.23.4) [40], using a sliding window approach with a window size of 4 nucleotides, removing regions with mean quality less than Q20, and trimming residual adapter sequences. Quality-filtered reads from each sample were then assembled using SPAdes (v4.0.0) with the transcriptome (--rna) settings [41,42]. Assembled contigs were classified against the NCBI core_nt database (downloaded in July 2025) [43] using BLASTn (v2.16.0) [44] with a maximum of 10 target sequences per query, and an e-value cutoff of 0.001. Additional post-BLAST filtering was performed using the Python polars library (v1.10.0), retaining only matches with at least 85% identity over 85% of the query length. Taxonomy was assigned based on the majority consensus of remaining target matches, using bitscore to break ties between multiple taxa.

For each sample, the abundance of each detection was approximated using the transcripts per million (TPM) measure. Reads were mapped to their respective assembly using HISAT2 (v2.2.1) [45] with sensitive settings, then compressed and sorted with samtools (v1.17). TPM values were calculated at the genus level across each taxon detected. Results were visualised using plotly (v5.18.0; Plotly, Montreal, QC, Canada) and UpSetR (v1.4.0) [46]. All figures were refined for publication using Inkscape (v1.4.4).

### 2.5. Passive Filtering Simulations and Sensitivity Assessment

Whatman 597 and PVDF filters (Table 1) were assessed for their performance in field conditions. An experiment was first conducted to assess the ability of filter membranes to capture viral particles following prolonged exposure to background organic material and other non-targeted eDNA from greenhouse irrigation water. Filters were placed into a 15 mL Falcon tube containing 10 mL of greenhouse irrigation water to allow circulating bioorganic matter to bind to the filter membrane. After an exposure time of 0, 2, or 23 h in the water, 100 µL of PepMV solution containing 100 million virus particles was added to the water for two hours to simulate a transient pulse of viral load.

To test the effect of the duration of virus exposure on test sensitivity, fresh filter membranes were placed into 15 mL Falcon tubes containing 10 mL greenhouse irrigation water, 100 µL of PepMV solution containing 100 million particles, 100 pg of luciferase RNA and 100 pg of KGMMV gBlock DNA. Filter membranes were collected for molecular testing following 2, 4, 8, and 24 h of exposure. Finally, to test the persistence of target binding, filters were exposed to the same spiked irrigation water conditions as above for 2 h and then washed with a continuous flow of irrigation water in a 12 L hydroponic flow system for 4, 18, 24, and 48 h, after which they were collected for testing. For all experiments, total nucleic acids were extracted using the CTAB method and tested for target presence using RT-qPCR.

### 2.6. Establishment of Capsid-Integrity PCR for Surveillance Testing

Experiments were conducted using purified PepMV virions, demonstrated to be infectious via bioassay, and with a high proportion of intact coat protein via a polyacrylamide gel. To simulate realistic detections in environmental samples, denaturation of virus particles was achieved using heat treatment. The stock PepMV solution containing 1 × 10^8^ particles per µL in glycerol storage buffer was diluted using DEPC-treated water to obtain 126 µL aliquots containing 1 × 10^6^ PepMV particles, then incubated at 25, 60, or 95 °C for 30 min, with a control sample incubated on ice for the same period. Following heating, samples were spiked with 14 µL of 2500 µM of either (a) DEPC water, (b) DMSO, or (c) platinum chloride (PtCl_4_) in DMSO. Samples were then incubated in a light-free environment for 30 min at room temperature.

Following incubation, RNA was extracted from sample solutions using the QIAamp Viral RNA Kit (Qiagen, Hilden, Germany) following manufacturer instructions but using a modified elution step. RNase-free water was used in place of AVE buffer, and elution was performed by adding 40 µL of RNase-free water, then incubating for 1 min and centrifuging. This elution process was then repeated to collect a total volume of 80 µL per sample. All samples were stored at −80 °C until further required. Detection of PepMV was performed using RT-qPCR.

To confirm that results obtained from capsid-integrity PCR (CI-PCR) were due to specific inactivation of non-viable PepMV RNA rather than full viral load, approximately 1 g of symptomatic leaf tissue was ground thoroughly in an extraction bag with 4 mL of 0.1 M phosphate buffer, pH 7.4, containing 5% PVP-40 (*w*/*v*) and 0.12% Na_2_SO_3_ (*w*/*v*). Aliquots were taken and treated at temperatures of 25, 60, 65, 68, and 95 °C to incrementally inactivate viral particles. For each temperature treatment, 1 mL of inoculum was administered to 10 healthy plants of *Nicotiana occidentalis*. Two leaves were inoculated per plant by first dusting them with silicon carbide (400 mesh particle size) (Sigma-Aldrich, St. Louis, MO, USA) as an abrasive. After rub inoculation, leaves were briefly rinsed with cold water. The indicator plants were maintained in glasshouse conditions and monitored for symptom development over a 2-week period. After 14 days, symptom severity on infected plants was assessed and scored for typical PepMV symptoms, and the presence of PepMV was confirmed by RT-qPCR.

Field sampling for CI-PCR trials was performed by placing 8 cm^2^ sections of Whatman 597 filter membranes into a custom 3D-printed housing structure using the ‘torpedo’ design described by Schang et al. [30] (Supplemental Figure S1). We deployed two torpedoes into each of four collection sumps at a field site previously known to harbour PepMV. Torpedoes were collected after three hours in the water system and stored on ice for return to the laboratory. Samples were stored at 4 °C overnight, then incubated with either a DMSO or PtCl_4_ solution, as described above, before nucleic acids were extracted using the CTAB extraction method and tested using qPCR. Statistical analysis of all qPCR data was performed using the scipy (v1.17.1) and statsmodels (v0.14.6) packages in Python (v 3.11.7) and the R statistical language (v 4.4.3) [47,48,49]. For comparing CI-PCR data obtained from PtCl_4_ and control (DMSO) field samples, a negative binomial generalised linear model (GLM) was fitted to target copy numbers extrapolated from a standard curve run during qPCR testing. The alpha parameter for each GLM was fitted using a method of moments approach starting from an alpha of 0.1 and adjusting until the delta between the current and new alpha value was less than 1 × 10^−6^.

## 3. Results and Discussion

### 3.1. Establishment of Passive Sampling and Molecular Detection Methods

**Figure 1:**
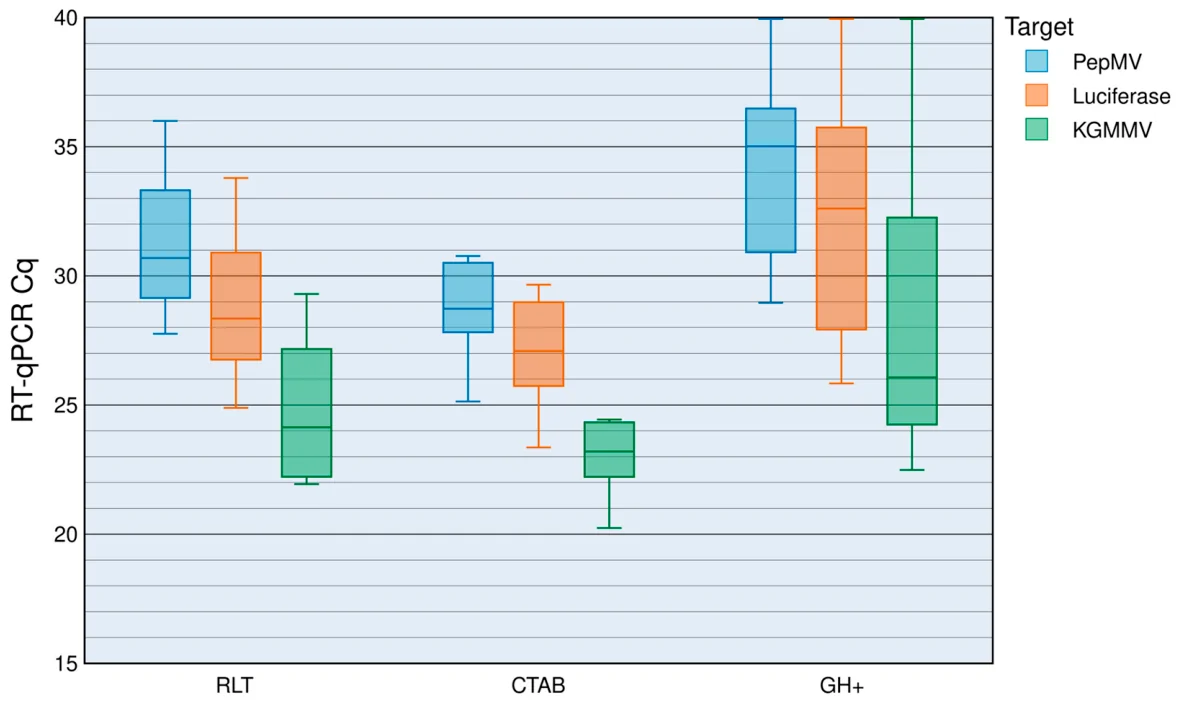
Box-and-whisker plot displaying a variation in PCR Cq values obtained from RT-qPCR assays for targets extracted using three different nucleic acid extraction protocols. Extraction protocol abbreviations are as described in the Section 2 but, briefly, refer to the RNeasy Plant Mini Kit with RLT buffer (RLT), CTAB lysis buffer with the InviMag Plant DNA Mini Kit (CTAB), and guanidine hydrochloride lysis with the InviMag Plant DNA Mini Kit (GH+). Five replicates of RT-qPCR reactions were performed for each extraction/target combination. Figure produced using Plotly and Inkscape.

Comparative evaluation of five filter membranes and three extraction techniques was performed with the aim of recovering three target types from the filter, assessed using RT-qPCR. When considered across extraction methods, the CTAB method yielded the most efficient amplification (Figure 1) and consistent signal (Table 2) across all methods.

With the CTAB extraction method yielding lower average Cq values for each target, filter membranes were considered within the CTAB extraction results. Each filter membrane tested in this project makes use of different binding chemistry for adsorbing target molecules. In many cases, the technical specifics of the membrane are proprietary information and not accessible, but their behaviours are known in broad terms. For example, the Whatman FTA membrane (Table 1) chemically lyses target cells on contact and contains preservatives to stabilise nucleic acids for long periods without additional storage considerations. By contrast, the Brightstar+, Millipore MCE, and PVDF filter membranes do not contain stabilising agents but are hydrophilic and bind less protein than nylon or nitrocellulose membranes. Although there are several studies comparing the efficacy of filter membranes for virus capture against other materials [50,51], there exists no comprehensive comparison of filter membranes for *Potexvirus* recovery. We therefore tested five different filter membranes to identify the best-performing candidate.

**Figure 2:**
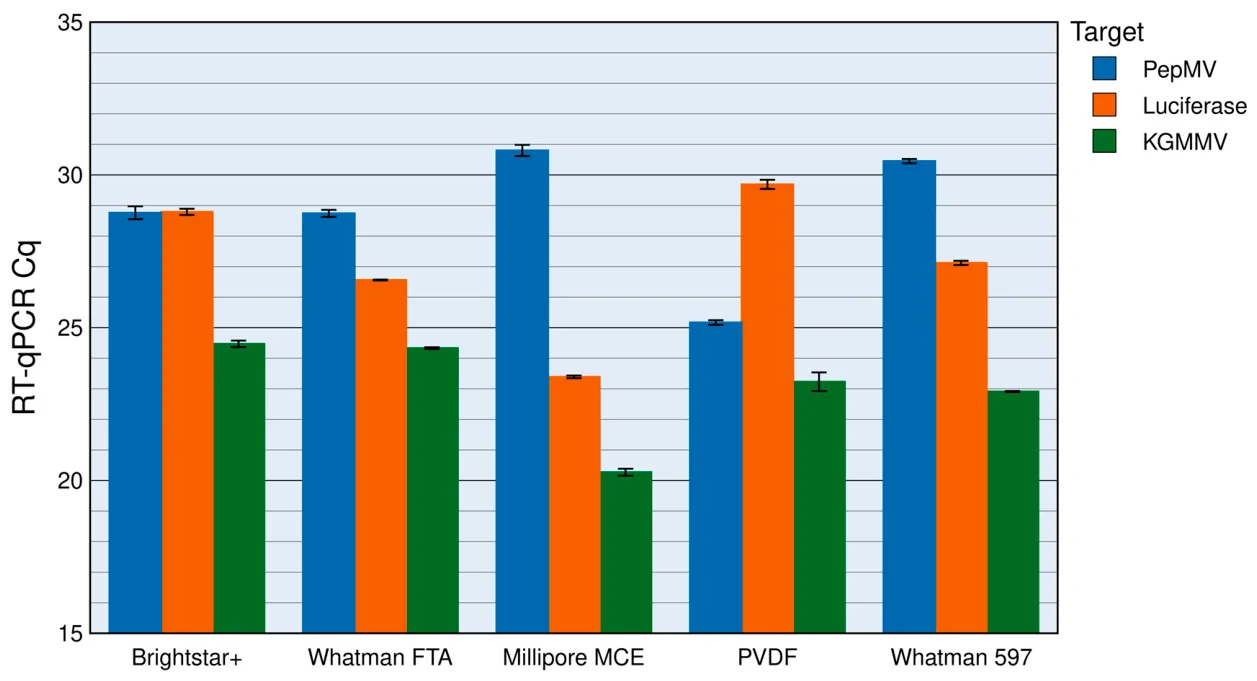
Average PCR Cq for detection via RT-qPCR when extracting filter membrane contents using the CTAB extraction method. Error bars show standard deviation for technical replicates of paired extractions. Figure produced using Plotly and Inkscape.

Across all three targets (PepMV, luciferase, and KGMMV), the Whatman 597 filter membrane yielded the most consistent results, although it did not produce the most sensitive RT-qPCR signal for any target (Table 2). By contrast, results from the PVDF filter membrane were the least consistent of the tested filters (Table 2) but demonstrated the most effective capture of PepMV particles compared with other methods (Figure 2). For these reasons, both the Whatman 597 and PVDF filter membranes were selected for trial under passive sampling conditions.

### 3.2. Identification of Ubiquitous Microorganisms for Use as Endogenous Control Material

It is common practice in a diagnostic setting to use a housekeeping gene from the host tissue as an internal control for assessing extracted nucleic acid PCR competency when testing for pathogenic organisms [52]. While an exogenous control, such as luciferase, can be used as a control for the nucleic acid extraction process, it cannot provide assurances about the inherent competency of the sampling. An additional endogenous control is needed for environmental samples. The usual targets for plant pathologists, such as NAD-5 and COX, are not always reliable when applied to irrigation water, as plant tissue may not be reliably shed into the water system or may be removed by filtering of larger particulates during water recycling. We collected samples from three commercial growing facilities, anonymised as Sites A, B, and C. Sampling was conducted twice from each site. Illumina MiSeq libraries from all six samples yielded a total of 18.9 M sequences, which were then assembled into approximately 382k transcripts (Table 3).

**Figure 3:**
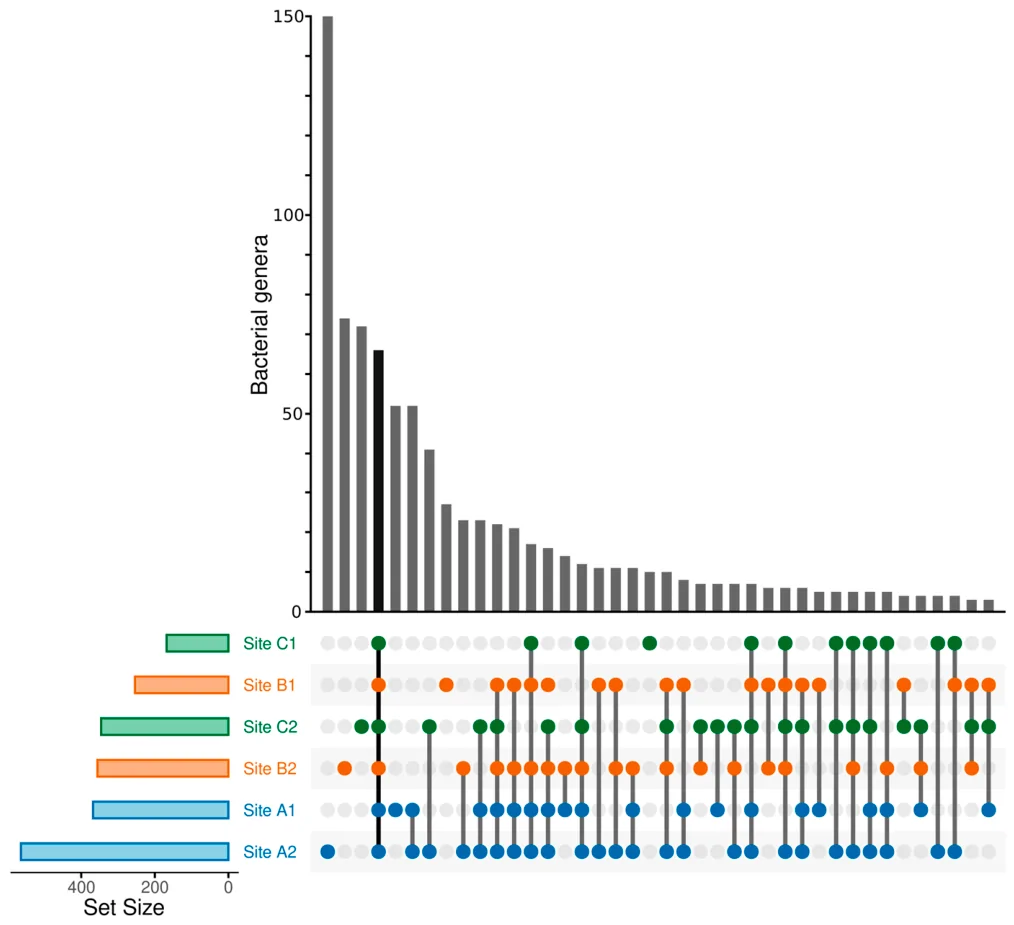
UpSet plot displaying the commonality of bacterial genera across all sites and time points following BLASTn classification and match filtering. A total of 66 bacterial genera were detected in all 6 samples (black). Figure produced using UpsetR and Inkscape.

**Figure 4:**
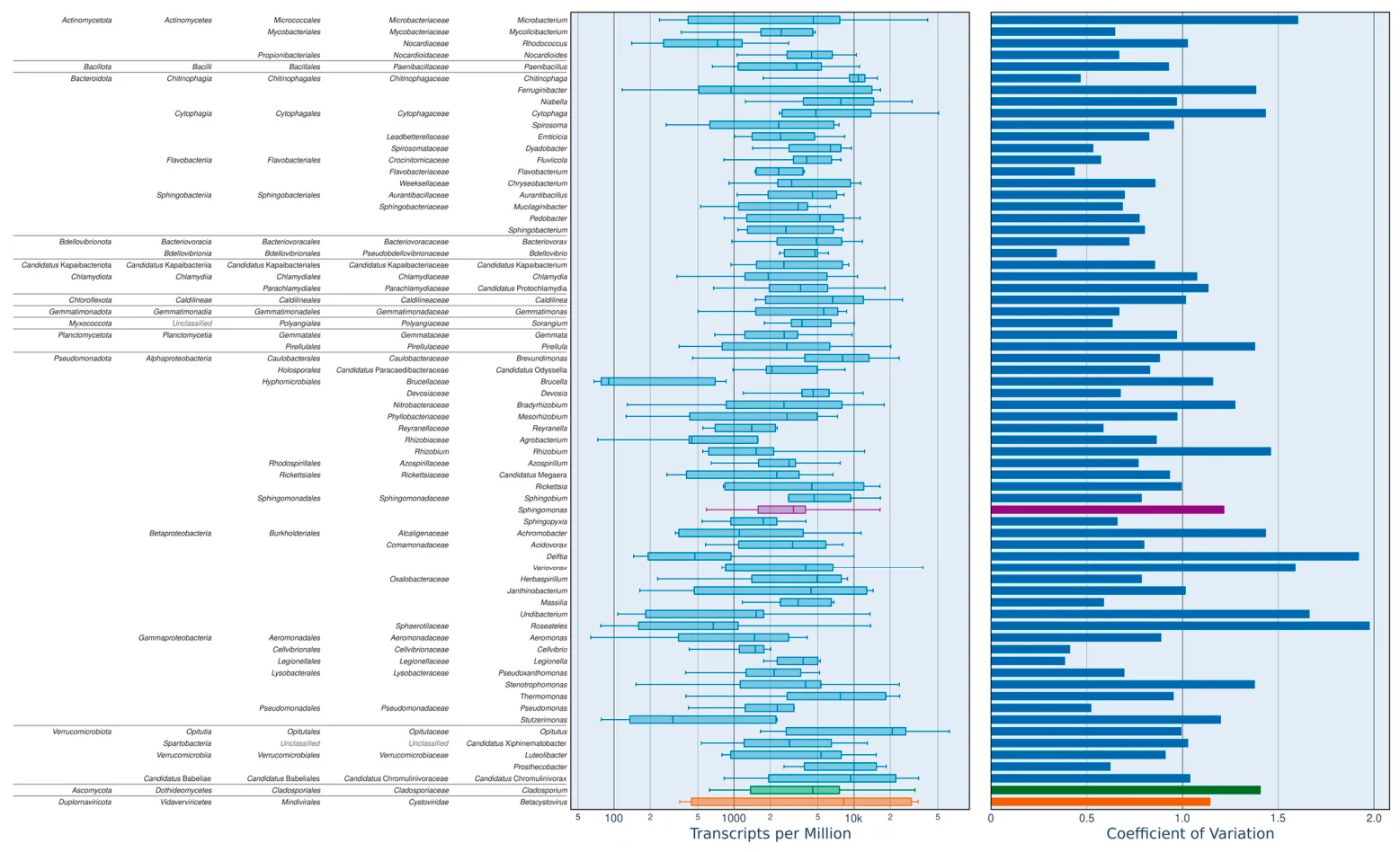
Expression and stability of signal from genera shared between all three sites and both time points surveyed by Illumina MiSeq, comprising 66 bacteria (blue, purple), 1 virus (green), and 1 fungus (orange). *Sphingomonas* (phylum *Pseudomonadota*) is highlighted in purple for identification purposes. TPM variation for each taxon of interest is displayed as box-and-whisker plots across all samples taken (**left**) along with the coefficient of variation for the 6 data points for each taxon (**right**). Figure produced using Plotly and Inkscape.

We identified a core set of 68 genera ubiquitous across all sampling sites and time points using BLASTn, 66 of which were bacterial (Figure 3). A single genus of virus and fungus was detected. PepMV was detected in both samples taken from Site A and Site C. These detections, and the absence at Site B, were validated with RT-qPCR (Table 3). We identified numerous bacterial genera as prevalent and consistently active, such as *Bdellovibrio*, *Cellvibrio*, *Flavobacterium*, *Sphingomonas*, and *Pseudomonas* (Figure 4). Based on these findings and their frequent detection in both natural [53,54,55] and engineered environments [56,57,58], we selected *Sphingomonas* as the target for an endogenous internal control PCR to be run alongside the PepMV assay.

### 3.3. Determining Viability of Protocol in Field Conditions

Based on these results, Whatman 597 and PVDF filter membranes were assessed for their virus recovery and retention under passive sampling conditions. We first tested the ability to capture and detect viral particles after prior accumulation of non-target biomass through exposure to irrigation water. This experiment was performed to simulate operating conditions under which PepMV is first introduced to a facility: target-free irrigation water passes over the passive sampling apparatus prior to the sudden, transient pulse of relevant virus particles encountering the filter. To simulate this scenario, PVDF and Whatman 597 filters were immersed in greenhouse irrigation water and exposed to PepMV. We then extracted total nucleic acids from the filter membranes and tested them for PepMV, luciferase, and *Sphingomonas* by qPCR.

**Figure 5:**
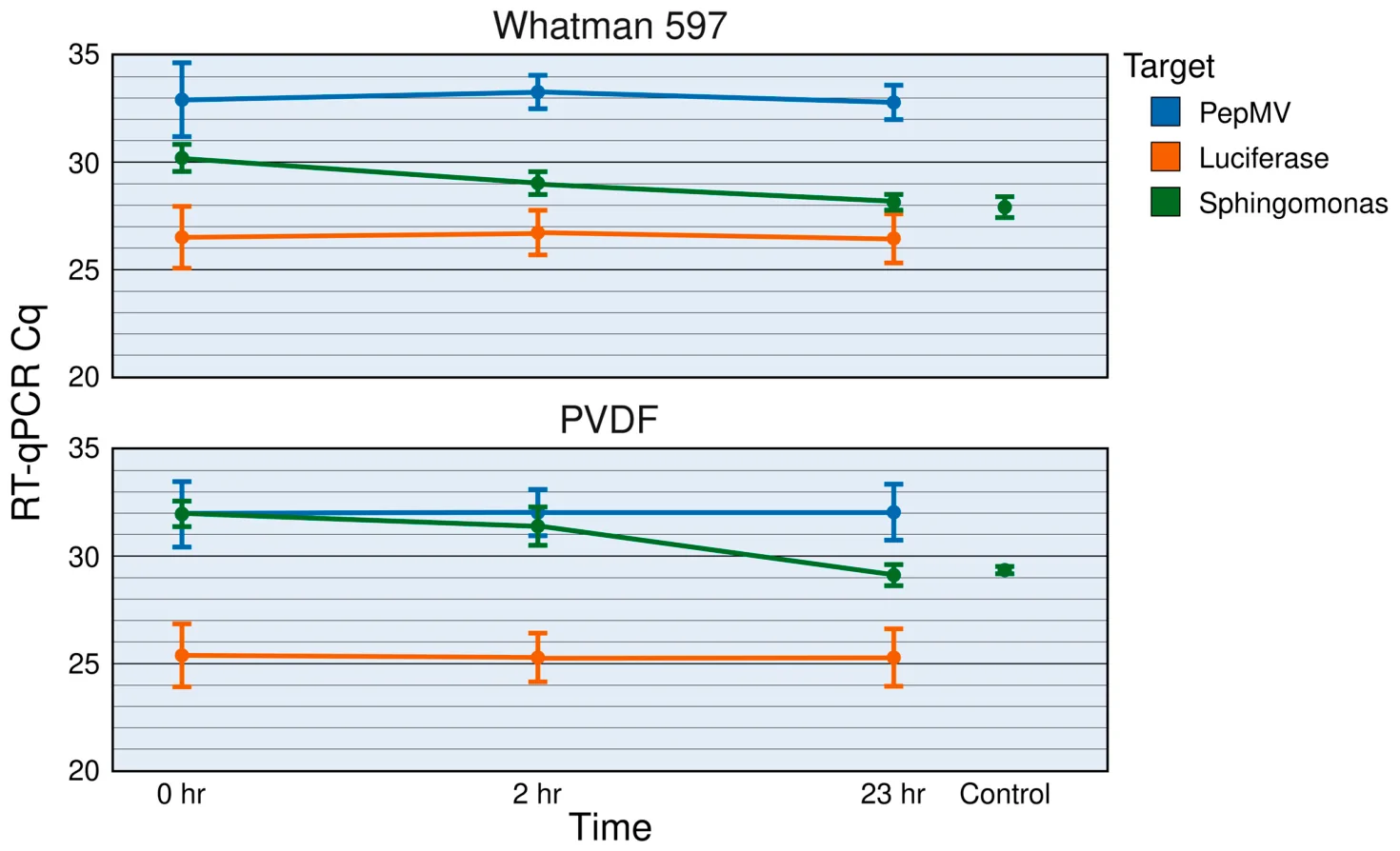
Variation in qPCR results for each target over prolonged exposure to irrigation water. Far right data point demonstrates *Sphingomonas* detection in control samples that were not spiked with PepMV or luciferase target molecules. Error bars denote standard deviation calculated across 4 reactions per time point and target. Figure produced using Plotly and Inkscape.

For each molecular target, the distribution of qPCR Cq values obtained for each time point was tested for normality using the Shapiro–Wilk test, then assessed for differences using one-way ANOVA. Statistically significant differences were not observed in the Cq values obtained from either PepMV or luciferase across irrigation water exposure times, indicating that the filters did not saturate with background nucleic acids or other materials. A reduction in average Cq thresholds for *Sphingomonas* was detected for both filter membranes with longer exposure times across the experiment (PVDF: F = 23.25, *p* < 0.001; Whatman: F = 16.49, *p* < 0.001). We performed pairwise comparisons between time points using *t*-tests to identify statistically significant differences between both time points for Whatman filters, but only between 2 and 23 h of exposure for PVDF filters (Table 4; Figure 5). These data show that biomass suspended in the water column accumulates on the filter membrane over time. This accumulation of biological material leads to a higher load of *Sphingomonas* DNA and an earlier detection in qPCR testing. However, over this period, the filters are not saturating to the point of being unable to collect viral particles, and the background portion of the nucleic acid yield during extraction is not sufficiently vast to dilute the viral signal to a degree where sensitivity is affected.

**Figure 6:**
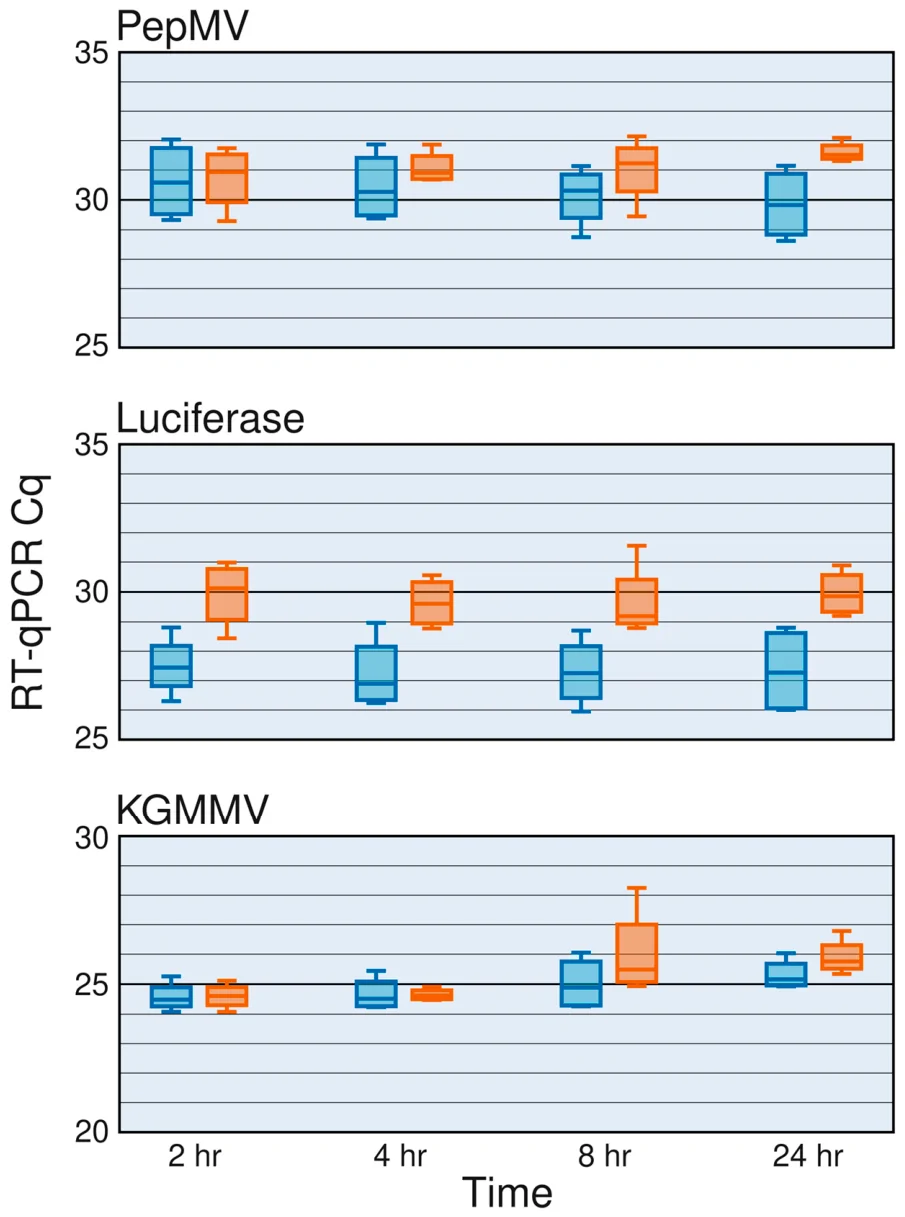
Box-and-whisker plots displaying variation in Cq signals obtained from RT-qPCR testing for RNA targets across four replicates following prolonged immersion of PVDF (blue) and Whatman 597 (orange) filter membranes in spiked irrigation water. Variation in Cq signal between time points for each filter membrane was not statistically significant when tested by ANOVA. Figure produced using Plotly and Inkscape.

Having established that filters retain the capability to capture viral particles following prolonged exposure to the background organic material expected within a commercial irrigation system, we next sought to test how well viral particles would accumulate over prolonged exposure. This experiment aims to identify a suitable interval for sample collection. Fresh filter membranes were incubated in irrigation water spiked with PepMV particles and a luciferase control for 2, 4, 8, and 24 h prior to collection. Results from RT-qPCR testing for the targets demonstrated minimal variation in detection across the duration of the experiment (Figure 6). Data were tested by one-way ANOVA, which confirmed that exposure time to viral particles did not have a statistically significant effect on RT-qPCR signal strength for PepMV detection (PVDF: F = 1.11, *p* = 0.39; Whatman: F = 2.47, *p* = 0.10). We suggest that the PepMV and luciferase targets were bound early in the experiment, potentially depleting the reservoir, leading to no significant improvement in detection over time. We compared the RT-qPCR signal strength between filter membranes for each target at each time point using two-sided *t*-tests for each target at each time point. No statistically significant difference in signal strength was observed between filter membranes at any time point once correction for multiple tests was performed (Supplemental Table S1).

Finally, we tested the retention of virus particles when ‘washed’ with virus-free irrigation water after virus exposure. Such an event may occur in growing facilities as diseased plants are identified and destroyed before identification of the causative agent can be made, or if different irrigation sources feed into a single outlet. Fresh filter membranes were inoculated in virus-spiked irrigation water, after which target-free irrigation water was washed over the membranes, with control samples taken prior to washing. Filter membranes were collected and tested for the presence of PepMV using RT-qPCR. All samples were positive for the virus, and no significant difference in RT-qPCR signal was detected across time points by ANOVA for PVDF (F = 1.11, *p* = 0.39) or Whatman 597 (F = 2.47, *p* = 0.10) filter membranes up to 48 h following particle adsorption.

We concluded from these experiments that both filter membranes were sufficient for field application. However, the PVDF filter requires methanol exposure as an activation step prior to use within the torpedo sampling tube. While the step is not particularly onerous, it does add a point of complexity and chemical handling that is not required by the Whatman 597 filter membrane. In contrast with our initial laboratory experiments (Figure 2), the PVDF filter membrane provided no advantage in virus sensitivity compared with the Whatman 597 filters. We recommend the Whatman 597 filter membrane for practical applications as it is cheaper and simpler to use and does not impact the diagnostic assay in any way.

### 3.4. Evaluation of PtCl_4_ Efficacy for Removal of Inactivated Virus RNA

**Figure 7:**
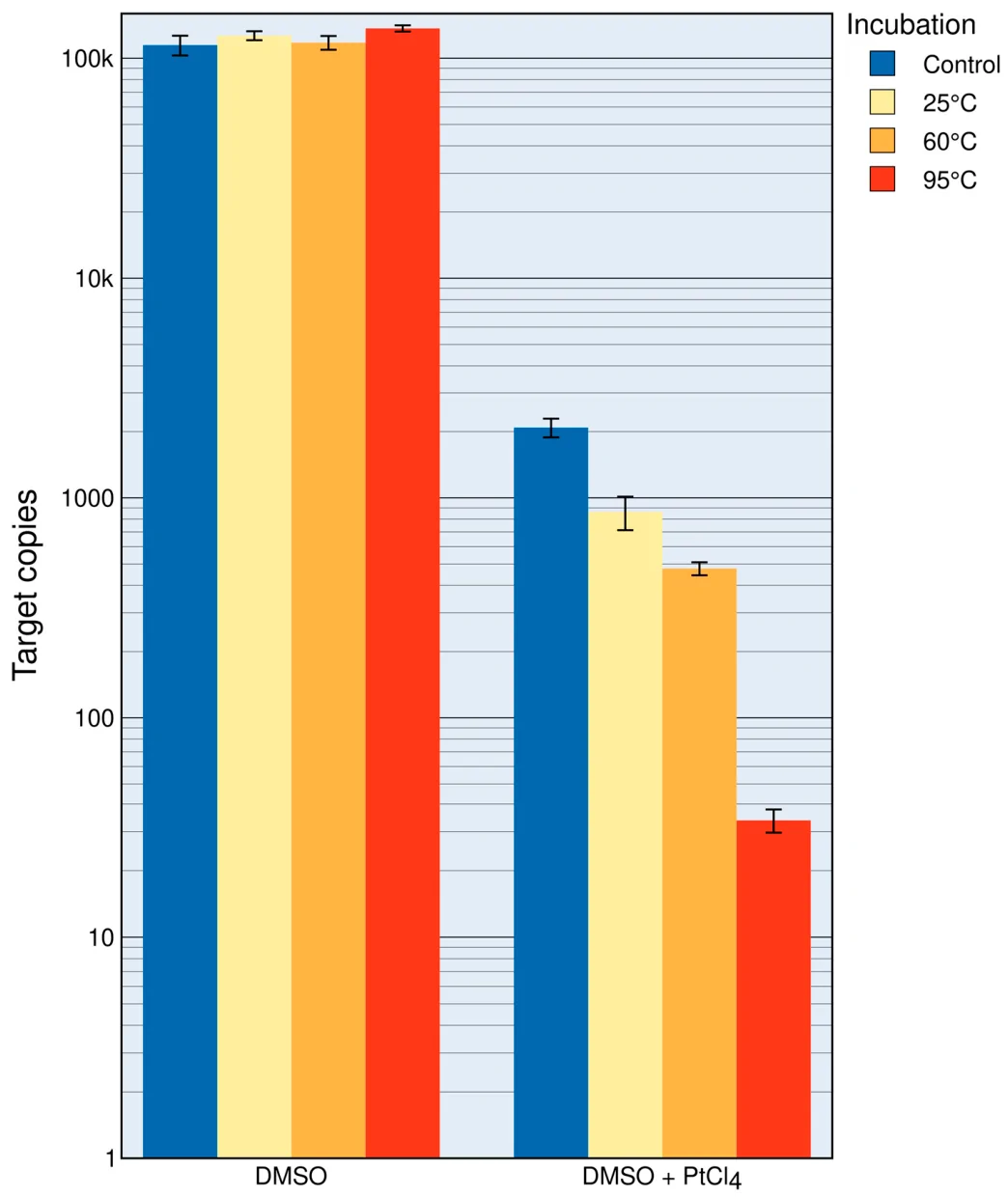
Estimated number of PepMV RNA targets in sample obtained through RT-qPCR compared to a reference standard. Samples were heat-treated to induce particle denaturation, then incubated with DMSO or DMSO + PtCl_4_ prior to RT-qPCR against a reference standard to calculate the number of target copies following treatment. Error bars denote standard deviation calculated from paired replicates. Figure produced using Plotly and Inkscape.

To account for the high likelihood of non-viable virus particles circulating within greenhouse water, protocols to differentiate between circulating free RNA and intact virions were developed. Based on previous reports of the high efficacy of PtCl_4_ in differentiating viable and non-viable SARS-CoV-2 particles [59], this method was chosen to deplete free circulating RNA without destroying intact and viable virions prior to nucleic acid extraction and RT-qPCR testing. Consistent with previously reported results for SARS-CoV-2, incubation with PtCl_4_ significantly decreased the availability of PCR-competent RNA in all treatments, with a more pronounced reduction observed at higher temperatures (Figure 7). Heat treatment controls without PtCl_4_ (DEPC or DMSO) had no meaningful impact on PCR-competent RNA, confirming that the target RNA was thermostable and that RT-qPCR signal reduction was due to PtCl_4_ incubation specifically. Healthy plants of *N. occidentalis* were inoculated with PepMV-infected plant sap that had undergone heat treatment, and after 14 days, they were assessed for the presence of PepMV symptoms. At temperatures of 68 °C and 95 °C, no PepMV symptoms were observed, whereas strong PepMV symptoms were noted in the lower-temperature treatments of 25, 60, or 65 °C. Confirmatory testing with RT-qPCR revealed that infection had failed to establish in the 95 °C treatment group. These results indicate the tipping point where virus particle denaturation occurs, and it corresponds with the initial reduction in viral load detected following PtCl_4_ treatment, where treatments at 25 °C and 60 °C yield an approximately 2- and 4-fold reduction in viral particles (Figure 7) before the signal drops by 2 orders of magnitude for the 95 °C treatment.

To demonstrate the applicability of this technique in the field, we deployed passive sampling torpedoes containing Whatman 597 filter membranes at four growth units around Site C (Table 3). Two torpedoes were deployed to each unit and recovered after 3 h of immersion in the water system. We performed CI-PCR for both PepMV and *Sphingomonas*, with either PtCl_4_ treatment or a control treatment in DMSO. The targets were detected in all samples, with a high variance observed between samples. We observed a trend of decreased target counts in the PtCl_4_-treated samples compared with their DMSO equivalents. A negative binomial generalised linear model was applied to the data to test the statistical significance of these decreased signals, but the difference was not statistically significant for either PepMV or *Sphingomonas* (Table 5). These results suggest that, although there may be some target nucleic acid in the water column from dead or inactive targets, the majority of signal obtained in each reaction originates from live bacteria and active (non-denatured) virus particles, which is consistent with the known prevalence of PepMV at this site.

### 3.5. Development of a Protocol Suite for Environmental Monitoring

We have established a set of protocols for the detection of infectious PepMV from greenhouse irrigation water based on passive sampling of the water system. Using HTS from samples taken across multiple sites and time points, we identified a potentially ubiquitous and stably active bacterial genus that can be used reliably as an endogenous control for confirming PCR competency when the virus is absent. We identified *Sphingomonas* as the candidate control organism for this work based upon its global prevalence across natural and engineered water systems and confirmed its presence by HTS and PCR testing. However, we acknowledge that this organism is not truly ubiquitous and may not be appropriate for all testing scenarios and advise researchers to robustly test any proposed control organism for suitability before deploying this method. Most of the 66 ubiquitous bacteria reported here, including *Sphingomonas*, possess a Gram-negative cell envelope (Figure 4). Such cells are typically easier to lyse during nucleic acid extraction than those with a Gram-positive peptidoglycan layer, yet this work did identify species from two Gram-positive phyla in the HTS data, namely Actinomycetota and Bacillota. Based on the extraction of HTS-competent DNA from our nucleic acid extraction method, we conclude that this method is suitable for finding a suitable bacterial replacement for *Sphingomonas* in water systems lacking this genus.

We have also provided data showing the effectiveness of the passive filtering approach and identified two effective filter membranes for capturing rare viral particles in the irrigation suspension. Finally, we developed a protocol to differentiate between active/intact viral particles and viral RNA from denatured particles.

When presenting the information above, we detected no difference in results obtained from either the PVDF or Whatman 597 filter membranes. In practice, we believe that the Whatman 597 filters will be more commonly used. This is due to both their cheaper retail cost and simpler and safer handling protocol. PVDF membranes require a brief immersion in methanol prior to use. This treatment is a minor consideration for research applications but, when applied at bulk in field settings, constitutes both a chemical hazard and fire risk. While it is possible to minimise both hazards with an operating procedure, our data find no significant benefit to the use of PVDF, which has been designed with specific chemical and electrophysical binding properties, over the simpler Whatman 597 filter membranes. We therefore believe that, for an organisation applying a risk management system such as the NIOSH Hierarchy of Controls, the Whatman 597 filter membranes are a more suitable product.

### 3.6. Final Considerations and Future Directions

The most significant unknown in the data we have presented is the maximum duration at which the passive sampling apparatus can be deployed before the filter is saturated with non-target nucleic acids to the point that molecular detection via PCR becomes unreliable. The benefit of the passive sampling system over a single capture of the water column is that the filter can be left unmonitored over the sampling period and requires minimal processing in the lab. However, while we have shown that PepMV particles can be detected up to 24 h after exposure, there was a trend of declining Cq in *Sphingomonas* qPCR over the period (Figure 5). The most likely interpretation of this phenomenon is the gradual accumulation of *Sphingomonas* biomass on the filter membrane. Over a prolonged period, this increasing load of bacterial cells entering the nucleic acid extraction phase and PCR testing could dilute the rarer PepMV target molecules to an undetectable level. The timeframe required for this effect, and whether it is a realistic concern for growing facilities, is unknown. We have also not tested the long-term stability of PepMV particles adhered to the filter membrane and cannot say with certainty how long they will be detectable if viruses in the water column were to vanish. Particle stability also has implications for the interpretation of this study’s downstream capsid-integrity assay. In our experience working in the biosecurity sector, however, we do not believe that such transient pulses of viruses are likely to occur in growing facilities. Once established in a greenhouse, economically important viruses such as PepMV cannot be eliminated without costly human intervention.

The question becomes one of how frequently the passive sampling system would be tested for virus particles. A sensible procedure would be to perform more frequent passive sampling testing when new consignments of plants are first established at the facility to ensure the earliest possible detection of viruses being introduced to the irrigation system. Once new plantings are established, it may be financially beneficial to reduce the frequency of testing as the risk of introducing pathogens to the growing system diminishes—assuming strict phytosanitary measures are in place. The balance of risk and benefit would be one for site operators to evaluate and determine based on their own operational experience and objectives.

## 4. Conclusions

We present here a combination of laboratory and field data demonstrating the development and application of a passive surveillance system for monitoring viral titre in irrigation water for early detection of plant pathogens before symptoms develop in crops. We have identified two filter membranes suitable for field deployment, each with slightly different behaviours but both sufficient for robust and sensitive detection of viral and bacterial RNA in the water system. Furthermore, using HTS, we have identified a core of ubiquitous organisms that can be used as endogenous control targets and deployed our own exogenous control to provide traceability across all points of failure in the diagnostic process. Finally, we provided evidence that the passive sampling method is robust to transient levels of viral titre in the water system and developed a simple method for eliminating background signal from non-viable RNA trapped in circulation. Determining the biological viability of a virus detected using molecular methods by using a rapid molecular assay in lieu of a lengthy bioassay provides critical information for the management of phytosanitary risk. Finally, we demonstrated the application of these techniques in three commercial growing facilities. Although we focused primarily on PepMV as a target organism, our battery of testing shows that both PCR- and HTS-competent RNA is recovered from a range of microorganisms, demonstrating the utility that this suite of techniques could be have beyond plant biosecurity in other fields such as ecological monitoring and human pathogen detection.

## Figures and Tables

**Table 1:** Filter membrane products tested during passive sampling experiments and their manufacturer information.

Label	Product	Product Number	Material	Manufacturer
Whatman FTA	QIAcard FTA PlantSaver cards	WB120065	Cellulose	Qiagen, Hilden, Germany
Whatman 597	Whatman Grade 597 Qualitative Filter Paper	10311862	Cellulose	Cytiva, Wilmington, DE, USA
Millipore MCE	MCE Membrane Filter	GSWP04700	Mixed cellulose esters	Merck KGaA, Darmstadt, Germany
BrightStar+	BrightStar-Plus Positively Charged Nylon Membrane	AM10100	Nylon	Applied Biosystems, Waltham, MA, USA
PVDF	PVDF Membrane Filter	HVWG04700	Polyvinylidene difluoride	Merck KGaA, Darmstadt, Germany

**Table 2:** Coefficient of variation (CV) for each target molecule by the nucleic acid extraction method and with the filter membrane used for collection. Filter membrane entries report results from the CTAB nucleic acid extraction method only.

Parameter	Variant	PepMV	Luciferase	KGMMV	Median CV
Extraction	RLT	0.09	0.10	0.11	0.10
	CTAB	0.07	0.08	0.07	0.07
	GH	0.11	0.15	0.21	0.15
Filter membrane	Brightstar+	0.14	0.15	0.08	0.14
	Whatman FTA	0.09	0.09	0.05	0.09
	Whatman 597	0.04	0.03	0.02	0.03
	Millipore MCE	0.03	0.09	0.10	0.09
	PVDF	0.15	0.06	0.23	0.15

**Table 3:** Read, assembly, and PepMV detection statistics for each sample site and time point assessed via Illumina MiSeq sequencing. RT-qPCR results for PepMV detection are reported as present/absent, as the relative abundance of virus particles between sites and time points is not a relevant consideration for diagnostic purposes.

Sample	Raw Reads	Filtered Reads (Singleton)	Transcripts	PepMV RT-qPCR	PepMV HTS
Site A1	2,135,453	2,082,254 (36,368)	36,357	+	+
Site A2	3,076,105	3,024,691 (48,312)	57,153	+	+
Site B1	3,809,356	3,764,653 (40,133)	67,953	-	-
Site B2	3,392,493	3,226,292 (105,076)	117,582	-	-
Site C1	3,269,103	3,005,125 (253,447)	57,119	+	+
Site C2	3,216,678	3,054,180 (113,787)	46,279	+	+

**Table 4:** *t*-test results comparing RT-qPCR detection results for *Sphingomonas* accumulation, as measured by Cq, across time points of the experiment. Two biological replicates were tested at each time point, with two technical replicates of each biological sample. Correction for multiple tests was performed using the Benjamini–Hochberg method to determine the false detection rate (FDR).

Membrane	Time Point 1	Time Point 2	*t* Statistic	*p*-Value	FDR
Whatman	0 h	2 h	2.84	0.029	0.043
Whatman	2 h	23 h	2.77	0.033	0.043
PVDF	0 h	2 h	1.07	0.326	0.326
PVDF	2 h	23 h	4.47	0.004	0.017

**Table 5:** Results of negative binomial GLM fitting to the detection of PepMV and *Sphingomonas* before and after PtCl_4_ treatment of field samples. The alpha parameter for each target was fitted using the method of moments approach. Rate ratio reports the proportion of molecules detected following PtCl_4_ treatment. A statistically significant decrease was not seen for either target.

Target	Fitted Alpha	Rate Ratio	95% CI	*p*-Value
PepMV	0.345	0.83	0.48, 1.43	0.51
*Sphingomonas*	0.098	1.00	0.75, 1.34	0.99

## Data Availability

The datasets presented in this article are not readily available due to confidentiality agreements signed with the commercial operators of the sampling sites. Requests to access the datasets should be directed to David W. Waite.

## Supplementary Materials

The following supporting information can be downloaded at https://www.mdpi.com/article/10.3390/v18080866/s1, Supplemental Figure S1. Selected images of torpedo filter cases with the casing open or closed by the removable lid. Supplemental Table S1. Statistical comparison of RT-qPCR signal strength (Cq) for the recovery of KGMMV, PepMV, and luciferase nucleic acids from PVDF and Whatman 597 filter membranes following exposure. Testing was performed using a two-tailed *t*-test, and normality of data was determined by the Shapiro–Wilk test prior to the *t*-test. Multiple test correction was performed using the Benjamini–Hochberg method and demonstrated no statistically significant difference in signal strength at any given time point following correction.

## Abbreviations

The following abbreviations are used in this manuscript:

eNA	Environmental nucleic acid
PepMV	Pepino mosaic virus
ToBRFV	Tomato brown rugose fruit virus
KGMMV	Kyuri green mottle mosaic virus
PCR	Polymerase chain reaction
qPCR	Quantitative PCR
RT-qPCR	Reverse-transcription qPCR
CI-PCR	Capsid-integrity PCR
HTS	High-throughput sequencing
TPM	Transcripts per million
ANOVA	Analysis of variance
COX	Cytochrome c oxidase (Complex IV)
NAD-5	NADH dehydrogenase subunit 5
