# Supplementary material for: Development and Validation of a Passive Surveillance System for Early Detection of Pepino Mosaic Virus in Commercial Greenhouse Facilities
Source: Viruses. 2026 Aug 7;18(8):866. doi: 10.3390/v18080866 (PMC13517131; doi:10.3390/v18080866)

Supplemental Figure S1. Selected images of torpedo filter cases with the casing open or closed by the removable lid.

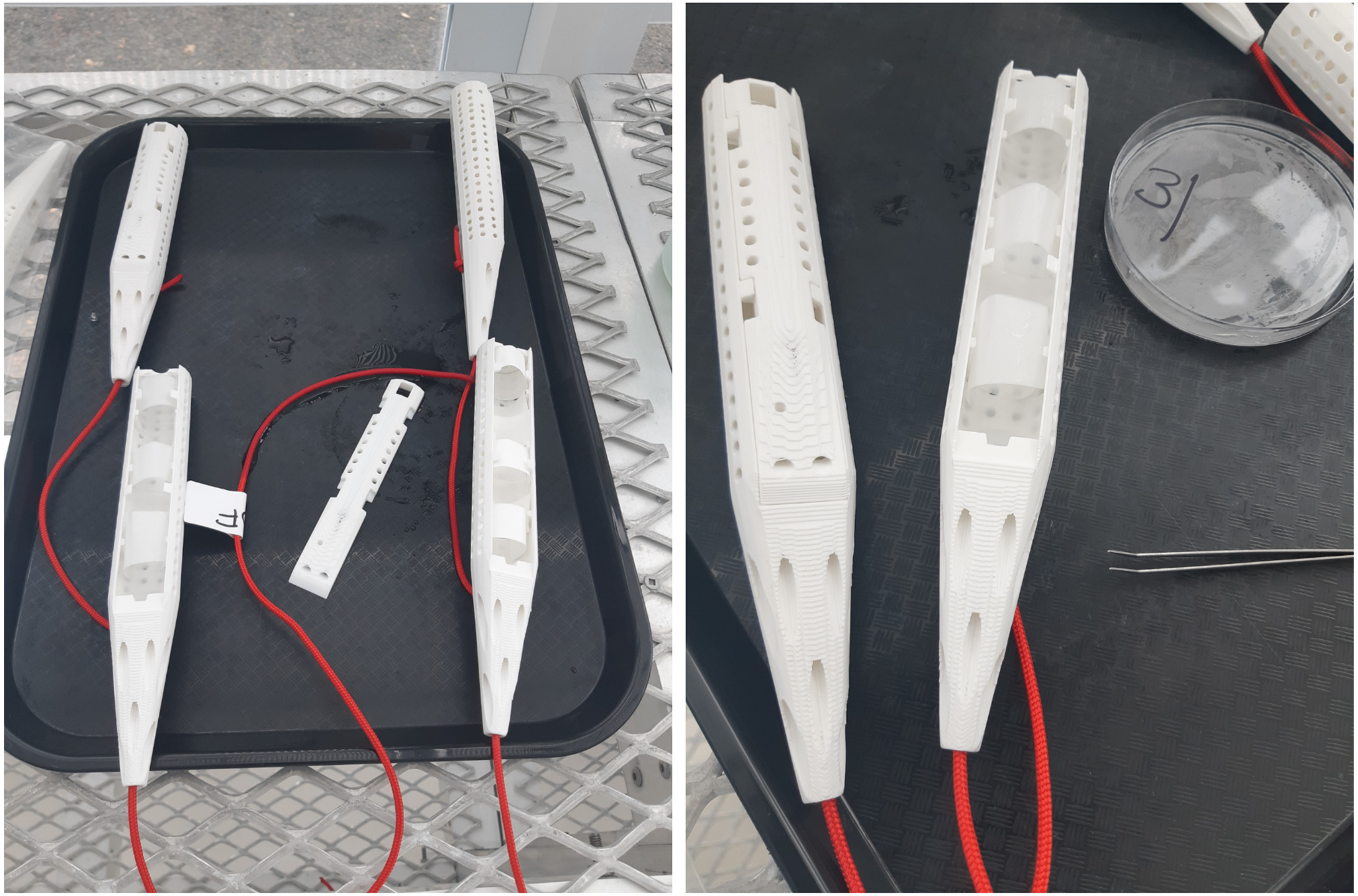

Supplement: Supplementary file 1 [file viruses-18-00866-s001.zip › Supplemental Figure S1.pdf]
